# Combined Blockade of Interleukin-1α and -1β Signaling Protects Mice from Cognitive Dysfunction after Traumatic Brain Injury

**DOI:** 10.1523/ENEURO.0385-17.2018

**Published:** 2018-04-13

**Authors:** Elizabeth A. Newell, Brittany P. Todd, Jolonda Mahoney, Andrew A. Pieper, Polly J. Ferguson, Alexander G. Bassuk

**Affiliations:** 1 Department of Pediatrics, University of Iowa Carver College of Medicine, Iowa City, Iowa; 2 Department of Psychiatry, University of Iowa Carver College of Medicine, Iowa City, Iowa; 3 Department of Neurology, University of Iowa Carver College of Medicine, Iowa City, Iowa; 4 Department of Free Radical and Radiation Biology Program, Department of Radiation Oncology Comprehensive Cancer Center, University of Iowa Carver College of Medicine, Iowa City, Iowa; 5 Department of Veterans Affairs, University of Iowa Carver College of Medicine, Iowa City, Iowa; 6 Pappajohn Biomedical Institute, Iowa Neuroscience Institute, University of Iowa Carver College of Medicine, Iowa City, Iowa

**Keywords:** Inflammation, interleukin-1, traumatic brain injury

## Abstract

Diffuse activation of interleukin-1 inflammatory cytokine signaling after traumatic brain injury (TBI) elicits progressive neurodegeneration and neuropsychiatric dysfunction, and thus represents a potential opportunity for therapeutic intervention. Although interleukin (IL)-1α and IL-1β both activate the common type 1 IL-1 receptor (IL-1RI), they manifest distinct injury-specific roles in some models of neurodegeneration. Despite its potential relevance to treating patients with TBI, however, the individual contributions of IL-1α and IL-1β to TBI-pathology have not been previously investigated. To address this need, we applied genetic and pharmacologic approaches in mice to dissect the individual contributions of IL-1α, IL-β, and IL-1RI signaling to the pathophysiology of fluid percussion–mediated TBI, a model of mixed focal and diffuse TBI. IL-1RI ablation conferred a greater protective effect on brain cytokine expression and cognitive function after TBI than did individual IL-1α or IL-1β ablation. This protective effect was recapitulated by treatment with the drug anakinra, a recombinant naturally occurring IL-1RI antagonist. Our data thus suggest that broad targeting of IL-1RI signaling is more likely to reduce neuroinflammation and preserve cognitive function after TBI than are approaches that individually target IL-1α or IL-1β signaling.

## Significance Statement

Traumatic brain injury is a leading cause of death and disability. Secondary signaling cascades, including neuroinflammation, trigger progressive neuronal injury after TBI. Interleukin-1 (IL-1) is a major driver of the neuroinflammatory response to brain injury, but the impact of individual IL-1α or IL-1β blockade on TBI outcome had not been evaluated. Using genetic and pharmacologic approaches, we dissected the contributions of IL-1α, IL-β, and IL-1RI to the pathophysiology of fluid percussion–mediated TBI. We show that genetic ablation of IL-1RI conferred the greatest benefit after TBI. Moreover, the beneficial effects were recapitulated by treatment with an IL-1RI antagonist. Given the need for the development of effective therapies that mitigate neurodegeneration after TBI, this is a timely, relevant area of study.

## Introduction

Traumatic brain injury (TBI), a leading cause of death and disability that affects upwards of 3 million Americans annually, is unfortunately characterized by a paucity of treatment options that are mainly limited to supportive care (Maas et al., 2008; Taylor et al., 2017). After a TBI, secondary signaling cascades, including robust neuroinflammation (Simon et al., 2017), trigger progressive neuronal dysfunction and neuronal death. These processes are additive to the initial tissue destruction at the time of physical injury. Because secondary injury pathways are executed in the hours to days after trauma, a broad therapeutic window exists during which progressive brain injury may be able to be prevented through therapeutic interruption of these secondary pathways. Neuroinflammation is a robust potential therapeutic target during this critical time window after a TBI.

A major component of the neuroinflammatory response to brain injury is release of interleukin-1 (IL-1), the prototypical pro-inflammatory cytokine that binds to the type 1 IL receptor (IL-1RI). After TBI, there is rapid activation of resident astrocytes and microglia, as well as infiltration of peripheral leukocytes, all of which may produce IL-1 cytokine. The primary receptor for IL-1 cytokine, IL-1RI, is also present on a variety of cell types, including both CNS and peripheral immune cells. Upon IL-1 binding to IL-1RI, a multitude of downstream inflammatory events are initiated. While the exact mechanisms are unknown, IL-1-induced neuronal injury has been extensively observed after excitotoxicity, ischemia, and TBI (Allan et al., 2005). Notably, IL-1 in the brain exists in two isoforms, IL-1α and IL-1β, which both increase rapidly after TBI (Griffin et al., 1994; Fan et al., 1995; Hutchinson et al., 2007). Although these two related cytokines signal through a common receptor (IL-1RI), IL-1α and IL-1β have distinct functions in sterile inflammation (Rider et al., 2011). For example, in a model of hypoxia-induced systemic inflammation, IL-1α release occurred earlier than release of IL-1β, was essential for initiation of leukocyte infiltration, and was specifically involved in neutrophil recruitment. By comparison, the later IL-1β response exhibited a greater role in propagation of inflammation and macrophage recruitment (Rider et al., 2011). In addition to distinct functions of IL-1α and IL-1β in systemic inflammation, different preclinical models of neurodegeneration also display different sensitivities to IL-1α or IL-1β blockade. After ischemic brain injury, for example, combined blockade of both IL-1α and IL-1β is necessary for maximal protection, whereas neuroprotection after spinal cord injury is achieved by selective IL-1α, but not IL-1β, blockade (Boutin et al., 2001; Bastien et al., 2015). Despite the magnitude and therapeutic potential of IL-1 signaling blockade after TBI, however, the impact of individual IL-1α or IL-1β blockade on TBI outcome has not previously been evaluated.

Because multiple IL-1 pathway-modulating drugs currently exist, this is a timely topic with respect to developing new treatments for patients suffering a TBI. For example, two IL-1β–specific inhibitors are FDA-approved for hereditary periodic fever syndromes (canakinumab and rilonacept) and systemic juvenile idiopathic arthritis (canakinumab), and one IL-1α inhibitor (MABP1) is currently in clinical studies in patients with sterile inflammation. More broadly, the IL-1R blocking drug anakinra is currently FDA-approved for rheumatoid arthritis and has also been studied for off-label use in other disorders with an inflammatory component, including stroke and TBI (Fisher et al., 1994; Emsley et al., 2005; Fleischmann et al., 2006; Helmy et al., 2014; Singh et al., 2014). Because of the disorder-specific roles of different components of IL-1 signaling in the brain, however, the specificity of cytokine modulation after TBI needs to be discerned to optimize the opportunity for patient care. We thus endeavored to dissect the relative magnitude of contribution of specific IL-1 pathway molecules on secondary injury after TBI.

Using separate lines of mice with genetic ablation of IL-1α, IL-1β, or IL-1RI, we interrogated the individual contributions of IL-1α and IL-1β, as well as their combined impact, on outcome after fluid percussion injury (FPI). We chose the TBI model of FPI because it recapitulates a common mixed injury in human TBI with both focal contusion and diffuse axonal injury throughout the brain (Narayan et al., 2002; Morales et al., 2005; Thompson et al., 2005; Dapul et al., 2013). Furthermore, as secondary injury mechanisms may differ between focal and diffuse injury, use of a mixed-injury model such as FPI importantly allows study of the impact of IL-1 signaling on both types of injury.

## Materials and Methods

### Animals

IL-1α–, IL-1β–, or IL-1RI–deficient male mice (IL-1α–/–, IL-1β–/–, or IL-1RI–/–) backcrossed on C57BL/6J genetic background were used. Generation of these mice has been previously described (Glaccum et al., 1997; Horai et al., 1998). Studies were conducted on adult mice, aged 2–6 months, with an average weight 25.5 ± 3.2 g. For behavioral studies, same-sex wild-type (WT) littermates were used as controls. In studies that did not involve behavioral assessment, WT littermates or age- and sex-matched C57BL/6J mice purchased from Jackson Laboratory were used as controls. Mice were housed in the Animal Care Facility at the University of Iowa under a 12-h light-dark cycle with *ad libitum* access to food and water. All mice were housed in the barrier facility until time of craniectomy, and then remained singly caged in a nonbarrier facility thereafter.

### Fluid percussion injury

Lateral FPI was performed as previously described (Alder et al., 2011). On the day before injury, mice underwent craniectomy. Animals were anesthetized with ketamine/xylazine (87 mg/kg ketamine and 12 mg/kg xylazine) via intraperitoneal injection. The head was then mounted in a stereotaxic frame, and a midline incision of the scalp was made for reflection of the skin and exposure of underlying skull. A 3-mm craniectomy was performed on the left parietal skull bone centered between lambda and bregma sutures and between lateral skull edge and sagittal suture, using a 3.0-mm OD handheld trephine (University of Pennsylvania Machine Shop). A modified Luer-Lock hub was placed surrounding the craniectomy site and secured with cyanoacrylate glue. The hub was further secured with methyl-methacrylate dental cement (Jet Acrylic Liquid mixed with Perm Reline/Repair Resin) surrounding the bottom portion of the hub. The hub was filled with sterile 0.9% saline, and a sterile IV cap was placed to prevent exposure of the underlying dura to the environment until time of FPI. The next day, mice underwent FPI. Mice received 3% inhaled isoflurane in an induction chamber before being transferred to a nose cone where IV cap was removed and any air bubbles in the hub were also removed. Once deeply anesthetized, mice were connected to the FPI device via 20-inch IV tubing and placed on their right side. A pendulum was released from an angle of 11–12 degrees against the fluid reservoir, generating a brief fluid pulse against the exposed dura. A Tektronix digital oscilloscope (TDS460A) was then used to measure the duration and peak pressure of the fluid pulse. After injury, mice were placed on their backs, and their righting time was measured as an indicator of injury severity. After righting, mice were re-anesthetized with 1%–2% isoflurane, the Luer-Lock hub was removed, and the skin incision was sutured closed. Mice receiving sham injury underwent identical treatment through connection to the FPI device, but were disconnected without triggering of the FPI device. After skin closure, anesthesia was discontinued and animals were placed in a heated cage until recovered and ambulatory. As we were interested in studying the impact of IL-1 on moderate to severe traumatic brain injury, mice were included only if righting reflex was >5 min (Thompson et al., 2005; Fenn et al., 2015; Schurman et al., 2017). Across all studies, the average righting time ± SEM was 8.27 ± 0.21 min, which corresponded to an average peak pressure delivered of 1.32 ± 0.01 ATM.

#### Study 1: Impact of IL-1 molecules on acute cytokine expression after FPI

Sham and FPI WT mice were compared to IL-1α–/–, IL-1β–/–, or IL-1RI–/– FPI mice. Mice were euthanized with an overdose of ketamine/xylazine at 6 h post-injury, followed by decapitation and rapid removal of their brains. Regional brain tissue was collected and snap frozen on liquid nitrogen for RNA extraction. IL-1α experiment: WT sham (*n* = 3), WT FPI (*n* = 6), IL-1α–/– FPI (*n* = 5); IL-1β experiment: WT sham (*n* = 3), WT FPI (*n* = 5), IL-1β–/– FPI (*n* = 8); and IL-1RI–/– experiment: WT sham (*n* = 3), WT FPI (*n* = 5), IL-1RI–/– FPI (*n* = 5). An additional group of WT sham (*n* = 3), WT FPI (*n* = 5) and IL-1RI–/– FPI (*n* = 6) mice were euthanized at 24 h post-injury and underwent the same procedures for 24-h RNA collection.

#### Study 2: Impact of IL-1 molecules on functional outcome after FPI

The following mice underwent sham or FPI procedures, followed by behavioral testing at several time points post-injury: IL-1α–/– (*n* = 12 sham, *n* = 21 FPI) and WT littermates (*n* = 11 sham, *n* = 17 FPI); IL-1β–/– (*n* = 11 sham, *n* = 15 FPI) and WT littermates (*n* = 9 sham, *n* = 14 FPI); IL-1RI–/– (*n* = 10 sham, *n* = 19 FPI) and WT littermates (*n* = 11 sham, *n* = 18 FPI). At 21 d post-injury, mice were euthanized and brains were collected for histologic analysis.

#### Study 3: Impact of anakinra treatment on cytokine expression 24 h after FPI

C57BL/6J mice were randomly assigned to receive two doses of intraperitoneal anakinra, 25 mg/kg (*n* = 7), or an equal volume of sterile 0.9% saline (*n* = 8) after FPI. Additional C57BL/6J mice underwent sham procedure and were untreated (*n* = 3). Anakinra (Sobi; 67 mg/100 ml) was diluted in sterile 0.9% saline. First treatment (anakinra or saline) was initiated within 30 min after FPI, and a second dose was given ∼12 h later. Twenty-four hours after FPI, mice were euthanized with an overdose of ketamine/xylazine and decapitated, and brains were rapidly removed. Regional brain tissue was collected and snap-frozen on liquid nitrogen for RNA extraction.

#### Study 4: Impact of anakinra treatment on functional outcome after FPI

C57BL/6J mice were randomly assigned to receive anakinra, 25 mg/kg IP (*n* = 10), or an equal volume of sterile 0.9% saline (*n* = 9) after FPI. Additional C57BL/6J mice underwent sham procedure and were untreated (*n* = 8). Mice were treated twice daily for 3 d after FPI. On day of FPI, twice daily treatment was initiated with first dose given within 30 min after FPI and second dose given 9 ± 1 h later. On the 2 d post-injury, anakinra or saline was given every 12 h. Test subjects underwent behavioral testing at various time points post-injury.

### Real-time PCR

Total RNA was extracted from sham or FPI brain regions using TRIzol (Invitrogen) as per the manufacturer’s instructions. RNA yield and purity were evaluated using a NanoDrop spectrophotometer. First-strand cDNA was synthesized with SuperScript III reverse transcriptase (Invitrogen). Amplified cDNAs were diluted 1:15 in ultrapure water and subjected to real-time PCR on an Applied Biosystems Model 7900HT with TaqMan Universal PCR Mastermix (Applied Biosystems), with the following probes: IL-1β (Mm00434228_m1), IL-1α (Mm00439620_m1), IL-6 (Mm00446190_m1), TNF-α (Mm00443258_m1), GAPDH (4308313). PCR reactions were conducted as follows: 2 min at 50°C, 10 min at 95°C, followed by 40 cycles for amplification at 95°C for 15 s, 60°C for 60 s. Biologic samples were run in triplicate. Genes of interest were normalized to endogenous control GAPDH. Data were analyzed using comparative cycle threshold method, and results were expressed as fold difference from sham controls.

### Immunohistochemistry and lesion volume analysis

Twenty-one days after FPI, mice were deeply anesthetized with ketamine/xylazine and transcardially perfused with 0.9% saline followed by 4% paraformaldehyde in 0.1 m PBS. Brains were extracted and immersed in the same fixative. The brains were then paraffin-embedded, and 6 μm coronal sections were cut with a rotary microtome. Sections were collected every 300 μm, mounted on Plus slides, and stained with hematoxylin and eosin for further analysis. An Olympic BX-61 microscope was used to image all sections. Lesion volume was calculated using sections encompassing the lesion from its anterior to posterior border, extending from approximately bregma –1.0 to −3.0 mm. Using ImageJ, the areas of the injured and uninjured hemisphere were measured on each serial section. Hemispheric volumes were then determined using volume equals sum of hemispheric areas multiplied by the distance between sections (300 µm). Impact of lesion on hemispheric volume loss was quantified by the following:

$$\%\text{ hemispheric volume loss}=\frac{\text{volume uninjured hemisphere}-\text{volume injured hemisphere}}{\text{volume uninjured hemisphere}}\times 100.$$

### Rotarod

Motor function was assessed using an accelerating rotarod (Columbus Instruments Rotamex-5). Each subject underwent 4 d of training before FPI, consisting of 3 trials/d with a 15-min rest interval between trials. The speed of the rotarod was accelerated from 4 to 40 RPM over 300 s, with an acceleration of 1.2 rpm/10 s. Latency to fall was recorded and averaged for the 3 trials. The highest average from pre-injury training was used as baseline performance. Mice were then tested on post injury days 1, 2, 3, and 7 using the same paradigm. The average latency to fall was recorded, and the percentage change from baseline performance was calculated. Test animals that were unable to remain on the accelerating rotarod for >60 s during training were excluded from analysis.

### Barnes maze

Cognitive function was assessed using the Barnes maze, which consists of a gray circular table 91 cm in diameter with 20 holes 5 cm in diameter evenly spaced around the perimeter. The table was brightly lit and open, motivating the test subjects to learn the location of the dark escape box located under one of the 20 holes. A black curtain surrounded the Barnes maze, and 4 equally spaced visual cues were hung from the curtain positioned around the table. AnyMaze video tracking software was used for data collection. Two weeks after sham or FPI, acquisition trials were conducted (4 trials/d) for 4 d, during which time an escape box was placed under the target hole. Each trial ended when the mouse entered the target hole or after 80 s had elapsed. Mice that did not locate the escape were guided to the target hole. All mice were allowed to remain in the escape box for 20 s. Average latency to the escape hole was recorded for each acquisition day. On day 5 of Barnes maze testing, a probe trial was conducted to assess hippocampal-dependent spatial memory. The escape box was removed from under the target hole and mice were placed in the maze for 60 s. Each mouse underwent 1 probe trial, during which the time spent in a 2-cm diameter zone around the target hole was recorded.

### Statistical analysis

Test animals were randomly assigned to treatment group. Lesion volume and behavioral analysis was performed by individuals blinded to genotype, injury, and treatment groups. Data were expressed as mean ± SEM. Data were analyzed with one-way or repeated-measures two-way ANOVA, followed by Fisher’s LSD or Dunn’s test for multiple comparisons. For multiday behavioral studies including rotarod and acquisition phase of Barnes maze, a split-plot experimental design was used and analyzed by repeated-measures two-way ANOVA. Treatment group was the between-subjects fixed factor, time was the repeated-measure fixed factor, and subject was the random factor. The interaction of time × treatment group was analyzed, as well as main effects of time and treatment. If significant effects were detected in the interaction or in the main effects, *post hoc* multiple comparison testing was done using Fisher’s LSD. Statistical analysis was done using GraphPad Prism, version 7.0. *p* < 0.05 was considered statistically significant. Tester was blinded to genotype and treatment.

### Study approval

All procedures performed in this study were in accordance with protocols approved by the Institutional Animal Care and Use Committee at the University of Iowa.

## Results

### Inflammatory cytokines are rapidly and diffusely up-regulated after FPI

**Figure 1.**
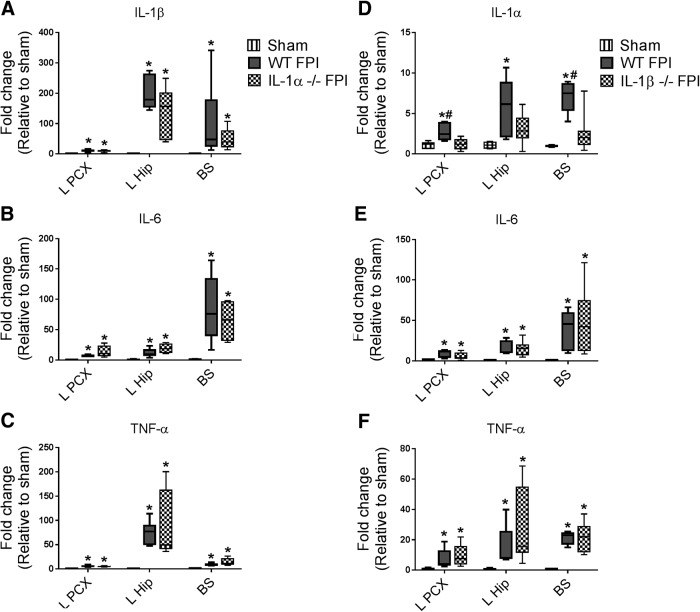
Acute inflammatory cytokine expression in IL-1α– and IL-1β–deficient mice after FPI. Expression of IL-1α, IL-1β, IL-6, and TNF-α were evaluated by qPCR in ipsilateral parietal cortex (L PCX), ipsilateral hippocampus (L Hip), and brainstem (BS) 6 h after FPI. ***A–C***, Cytokine expression was compared in WT sham (*n* = 3), WT FPI (*n* = 6), and IL-1α–/– FPI (*n* = 5) mice. ***D–F***, Cytokine expression was compared in WT sham (*n* = 3), WT FPI (*n* = 5), and IL-1β–/– FPI (*n* = 8) mice. Data are expressed as fold change in gene expression relative to sham and are presented as box-and-whisker plots; the box extends from 25th to 75th percentiles, the line represents the median, and the whiskers extend from smallest to largest value. One-way ANOVA with Fisher’s LSD or Dunn’s test for multiple comparisons. *, *p* < 0.05 compared with sham, #, *p* < 0.05 compared with IL-1β–/– FPI mice. The data are pooled from three (IL-1α) and two (IL-1β) independent experiments.

**Figure 2.**
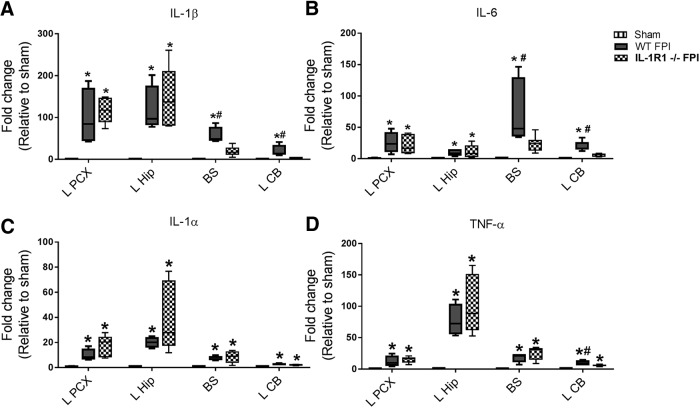
IL-1RI deficiency decreased cytokine expression in regions of diffuse injury 6 h after FPI. Expression of IL-1β (A), IL-6 (B), IL-1α (C), and TNF-α (D) was evaluated by qPCR in ipsilateral parietal cortex (L PCX), ipsilateral hippocampus (L Hip), brainstem (BS), and left cerebellum (L CB). Cytokine expression was compared in WT sham (n = 3), WT FPI (n = 4), and IL-1RI–/– FPI (n = 5) mice. Data are expressed as fold change in gene expression relative to sham and are presented as box-and-whisker plots; the box extends from 25th to 75th percentiles, the line represents the median, and the whiskers extend from smallest to largest value. One-way ANOVA with Fisher’s LSD or Dunn’s test for multiple comparisons. *, p < 0.05 compared with sham, #, p < 0.05 compared with IL-1RI–/– FPI mice. The data are pooled from two independent experiments.

Six hours after FPI, we identified a diffuse increase in IL-1α, IL-1β, TNFα, and IL-6 (Figs. 1 and 2) within parietal cortex, hippocampus, brainstem, and cerebellum, consistent with previous reports showing rapid upregulation of inflammatory cytokine expression after TBI (Fenn et al., 2015; Kumar et al., 2016). We have now additionally identified a broad increase in cytokine expression not only at the injury epicenter, but also in the brainstem and cerebellum, areas remotely located from the site of impact.

### Diffuse cytokine expression is blocked to a greater degree by IL-1RI ablation than by individual IL-1α or IL-1β ablation

IL-1 cytokines are major mediators of both systemic inflammation and neuroinflammation and lead to activation of several transcription factors that induce expression of hundreds of downstream genes, including cytokines and other pro-inflammatory mediators (Auron 1998; Basu et al., 2004; Weber et al., 2010). IL-1α and IL-1β also induce expression of their own genes in an autocrine manner (Basu et al., 2004; Weber et al., 2010). We therefore evaluated the impact of IL-1α, IL-1β, and IL-1RI signaling on inflammatory cytokine expression after FPI.

As described above, after FPI, we have demonstrated a rapid increase in inflammatory cytokine expression in various regions throughout the brain. In our experiments of IL-1α–deficient and WT mice, FPI resulted in increased expression of IL-1β, IL-6, and TNFα in ipsilateral cortex, ipsilateral hippocampus, and brainstem compared with sham subjects (Fig. 1*A–C*). IL-1α deficiency resulted in no difference in FPI-induced cytokine expression compared with WT mice in any brain region examined (Fig. 1*A–C*). In our experiments involving IL-1β deficient and WT mice, FPI again resulted in the expected increased cytokine expression throughout the brain, with increases of IL-1α, IL-6, and TNFα in ipsilateral cortex, ipsilateral hippocampus, and brainstem in WT FPI compared with sham subjects (Fig. 1*D*, *E*). Unlike in IL-1α–deficient mice, however, IL-1β deficiency resulted in mild impacts on inflammatory cytokine expression after FPI. Compared with WT FPI mice, IL-1β–deficient mice exposed to FPI had decreased IL-1α expression in the ipsilateral parietal cortex and brainstem, as well as a trend toward decreased IL-1α expression in the ipsilateral hippocampus (Fig. 1*D*). Finally, we evaluated the impact of IL-1RI deficiency on cytokine expression after FPI. In our experiments involving IL-1RI–deficient and WT mice, again as expected, FPI resulted in diffuse increases in inflammatory cytokine expression in WT FPI compared with sham subjects (Fig. 2*A–D*). In IL-1RI–deficient mice, however, we found the greatest magnitude of effect on FPI-induced cytokine expression, as multiple cytokines were decreased after injury at sites remote from impact (Fig. 2*A*, *B*). IL-1β and IL-6 expression were significantly decreased in the brainstem of IL-1RI–deficient mice compared with WT mice after FPI, but not in ipsilateral parietal cortex or ipsilateral hippocampus (Fig. 2*A*, *B*). To confirm the impact of IL-1RI deficiency on diffuse cytokine expression, we evaluated an additional region remotely located from the site of impact in IL-1RI–deficient mice, the cerebellum, and found that IL-1β and IL-6 were also decreased compared with WT after FPI (Fig. 2*A*, *B*). Thus, IL-1RI appears to play a major role in pathologic propagation of diffuse cytokine expression after TBI, with IL-1β having much less impact and IL-1α no role whatsoever.

### IL-1RI ablation accelerates resolution of cytokine expression 24 h after injury

**Figure 3.**
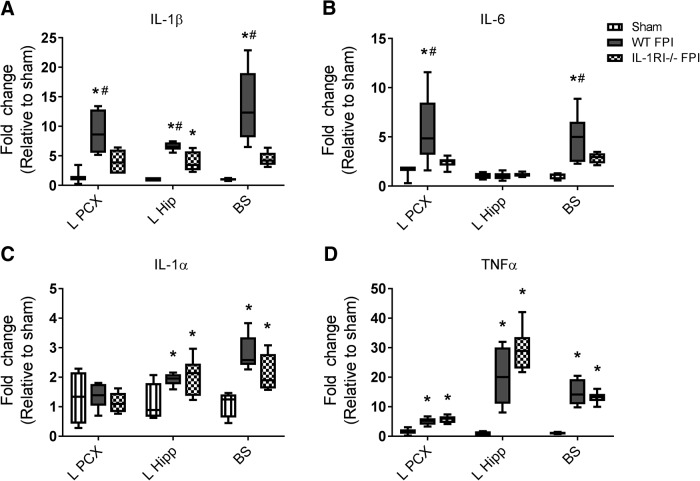
IL-1RI deficiency hastens resolution of IL-1β and IL-6 expression 24 h after FPI. Expression of IL-1β (A), IL-6 (B), IL-1α (C), and TNF-α (D) was evaluated by qPCR in ipsilateral parietal cortex (L PCX), ipsilateral hippocampus (L Hip), and brainstem (BS). Cytokine expression was compared in WT sham (*n* = 3), WT FPI (*n* = 5), and IL-1RI–/– FPI (*n* = 6) mice. Data are expressed as fold change in gene expression relative to sham and are presented as box-and-whisker plots; the box extends from 25th to 75th percentiles, the line represents the median, and the whiskers extend from smallest to largest value. One-way ANOVA with Fisher’s LSD for multiple comparisons. *, *p* < 0.05 compared with sham, #, *p* < 0.05 compared with IL-1RI–/– FPI. The data are pooled from two independent experiments.

As noted previously, at 6 h post-FPI, IL-1RI deficiency was associated with markedly decreased IL-1β and IL-6 expression in diffuse regions of the brain, but not in the regions of direct impact. At the site of TBI impact, necrotic cell death results in rapid release of endogenous pathologic signals, including ATP, HGMB1, and other potent stimulators of glial and immune cell cytokine expression (Gadani et al., 2015). While it would be unlikely that IL-1 pathway blockade could prevent the initial cytokine surge triggered at the injury epicenter by this wave of immediate necrotic cell death, we hypothesized that autocrine IL-1 signaling might propagate an ongoing inflammatory response throughout the brain by glia and immune cells. We thus tested whether blockade of IL-1RI signaling could hasten resolution of inflammation by examining cytokine expression 24 h after FPI in IL-1RI–deficient and WT mice (Fig. 3). Because the magnitude of 6-h cytokine suppression was greatest in IL-1RI–deficient mice, we focused only on this genotype for cytokine expression experiments conducted 24 h after FPI. In contrast to what we observed at the 6-h time point after FPI, there was decreased IL-1β and IL-6 expression in directly contused areas (ipsilateral parietal cortex and hippocampus), as well as areas remote from impact (brainstem), 24 h after injury. Specifically, IL-1β expression was decreased in ipsilateral parietal cortex, ipsilateral hippocampus, and brainstem of IL-1RI–deficient mice compared with WT. Furthermore, IL-1RI–deficient mice exhibited no increase in IL-1β expression above sham levels in the parietal cortex or brainstem (Fig. 3*A*). For IL-6, IL-1RI–deficient mice had decreased expression in ipsilateral parietal cortex and brainstem compared with WT after FPI. In the ipsilateral hippocampus, IL-6 was no longer increased in either WT or IL-1RI–deficient FPI mice, compared with sham mice (Fig. 3*B*). Similar to 6 h post-injury, there was no impact of IL-1RI ablation on IL-1α or TNFα expression (Fig. 3*C*, *D*).

### Ablation of IL-1α-, IL-1β-, or IL-1RI does not affect cortical lesion volume or sensorimotor function after FPI

**Figure 4.**
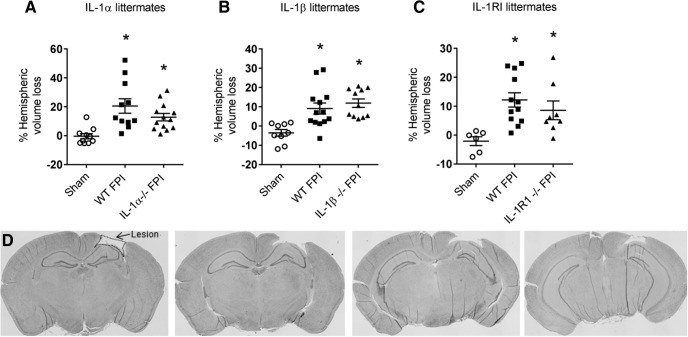
Tissue loss after FPI in in IL-1α–, IL-1β–, and IL-1RI–deficient mice. Quantification of cortical lesion volume 21 days post-injury. Data are expressed as percentage hemispheric volume loss compared with contralateral, uninjured hemisphere. ***A***, IL-1α littermates: WT sham (*n* = 10), WT FPI (*n* = 11), IL-1α–/– FPI (*n* = 13). ***B***, IL-1β littermates: WT sham (*n* = 9), WT FPI (*n* = 13), IL-1β–/– FPI (*n* = 11). ***C***, IL-1RI littermates: WT sham (*n* = 6), WT FPI (*n* = 12), IL-1RI–/– FPI (*n* = 9). ***D***, Representative images of hematoxylin and eosin–stained serial coronal sections identifying cortical lesion from a subject 21 days after FPI. Data are presented as mean ± SEM. One-way ANOVA with Dunn’s test for multiple comparisons. *, *p* < 0.05 compared with sham. The data are pooled from 4–8 independent experiments per IL-1 strain.

**Figure 5.**
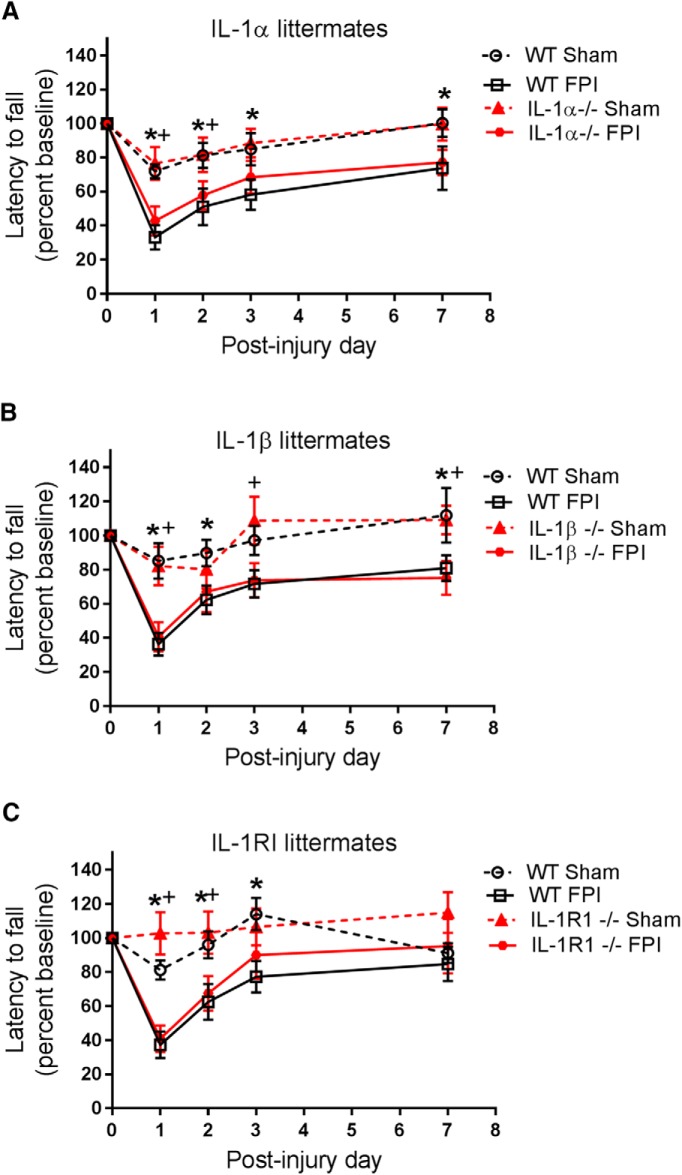
Sensorimotor impairment after FPI in IL-1α–, IL-1β–, and IL-1RI–deficient mice. Sensorimotor function was assessed in sham and FPI injured mice with an accelerating rotarod assay. Mice underwent pretraining for 4 d before injury to establish baseline performance. Testing was conducted on post-injury days 1–3 and 7. Latency to fall was recorded and normalized to pre-injury testing baseline. ***A***, IL-1α littermates: WT sham (*n* = 11), WT FPI (*n* = 17), IL-1α–/– sham (*n* = 12), IL-1α–/– FPI (*n* = 21). ***B***, IL-1β littermates: WT sham (*n* = 9), WT FPI (*n* = 14), IL-1β–/– sham (*n* = 11), IL-1β–/– FPI (*n* = 15). ***C***, IL-1RI littermates: WT sham (*n* = 11), WT FPI (*n* = 18), IL-1RI–/– sham (*n* = 10), IL-1RI–/– FPI (*n* = 19). Data are presented as mean ± SEM. Two-way RM ANOVA with Fisher’s LSD for multiple comparisons. *, *p* < 0.05 WT sham compared with WT FPI, +, *p* < 0.05 KO sham compared with KO FPI. The data are pooled from 7–10 independent experiments per strain.

After analyzing the impact of IL-1 blockade on brain cytokine expression, we assessed its impact on histologic and functional outcome. To ensure consistent injury across all animals, we recorded the peak pressure of every fluid pulse delivered, along with the time required for each subject to display an appropriate righting reflex. Across all three lines, consistent injury severity was demonstrated, as there were no differences between any of the IL-1 pathway–deficient mice and their WT littermates in either peak pressure delivered or time until righting. Assessment of lesion volume 21 d post-FPI also showed no differences between any of the IL-1 pathway–deficient mice and their WT littermates (Fig. 4). Because the lesion partially involves the sensorimotor cortex, it was not surprising that there were no differences between IL-1 pathway–deficient mice and their WT littermates in performance on the accelerating rotarod, a measure of sensorimotor function (Fig. 5). In experiments involving IL-1α WT and KO littermates, the interaction of time × treatment was not significant (*F*_12,212_ = 1.72, *p* = 0.07), but the main effects of time (*F*_4,212_ = 31.92, *p* < 0.0001) and treatment (*F*_3,53_ = 3.74, *p* = 0.02) were significant. *Post hoc* testing revealed differences between sham and FPI groups, but no difference between genotype groups on multiple days of testing. In experiments involving IL-1β WT and KO littermates, there was a significant interaction of time × treatment (*F*_12,180_ = 2.40, *p* = 0.007). *Post hoc* testing revealed differences between sham and FPI groups, but no difference between genotype groups after FPI. In experiments involving IL-1RI WT and KO littermates, there was a significant interaction of time × treatment (*F*_12,188_ = 3.50, *p* = 0.0001), but *post hoc* testing again revealed differences only between sham and FPI groups, with no difference between genotype groups after FPI (Fig. 5).

### IL-1RI ablation, but not individual IL-1α or IL-1β ablation, protects mice from cognitive deficits after FPI

**Figure 6.**
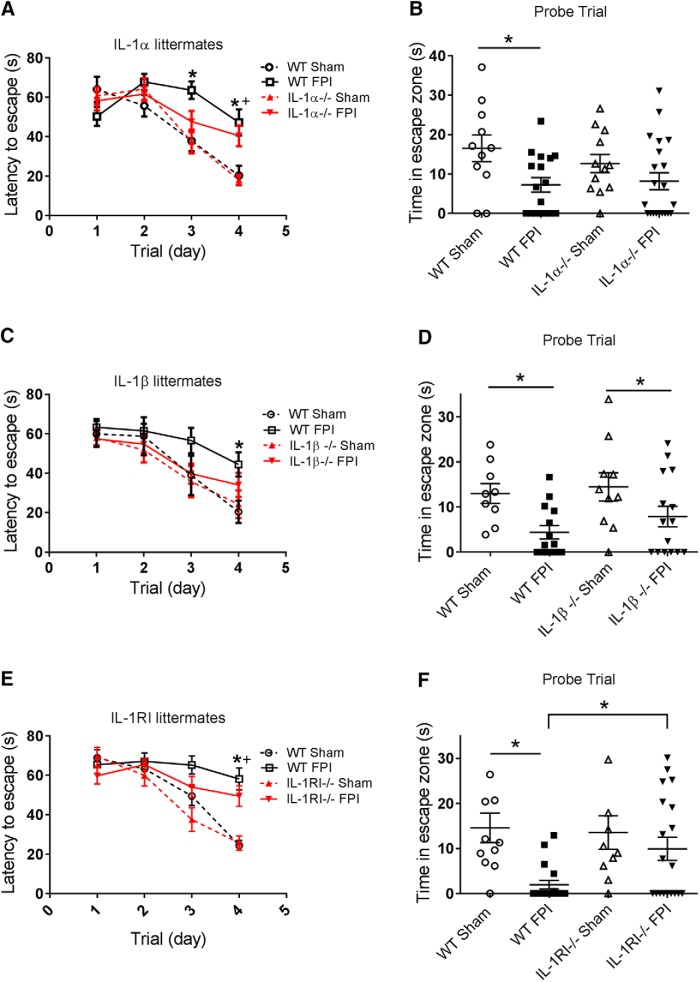
IL-1RI deficiency, but not individual IL-1α or IL-1β deficiency, resulted in improved cognitive function after FPI. The impact of IL-1α, IL-1β, and IL-1RI deficiency on spatial learning and memory after FPI was assessed using the Barnes maze. Mice exposed to sham or FPI injury underwent Barnes maze testing beginning 2 wk post-injury. Spatial learning was assessed with 4 d of spatial acquisition training. A probe trial on day 5 of testing assessed short-term reference memory. ***A***, ***B***, IL-1α littermates: WT sham (*n* = 11), WT FPI (*n* = 17), IL-1α–/– sham (*n* = 12) IL-1α–/– FPI (*n* = 21). ***C***, ***D***, IL-1β littermates: WT sham (*n* = 9), WT FPI (*n* = 14), IL-1β–/– sham (*n* = 11), IL-1β–/– FPI (*n* = 15). ***E***, ***F***, IL-1RI littermates: WT sham (*n* = 11), WT FPI (*n* = 18), IL-1RI–/– sham (*n* = 10), IL-1RI–/– FPI (*n* = 19). Data are presented as mean ± SEM. Acquisition training, two-way RM ANOVA with Fisher’s LSD for multiple comparisons. *, *p* < 0.05 WT sham compared with WT FPI, +, *p* < 0.05 KO sham compared with KO FPI, #, *p* < 0.05 KO FPI compared with WT FPI. Probe trial, one-way ANOVA with Fisher’s LSD for multiple comparisons. *, *p* < 0.05. The data are pooled from 7–10 independent experiments per strain.

Barnes maze testing was initiated 2 wk after FPI to quantify cognitive function. Mice first underwent 4 d of training, during which spatial learning was evaluated. They were then challenged with a probe trial on day 5 to evaluate short-term spatial memory retention. In the Barnes maze training experiments involving IL-1α WT and KO littermates, there was a significant interaction of time × treatment (*F*_9,171_ = 5.093, *p* < 0.0001). *Post hoc* testing revealed differences between sham and FPI treatment groups on days 3 and 4 of training (latency to escape day 3 WT sham mean = 37.9 vs. WT FPI mean = 63.6, *p* = 0.007; day 4 WT sham mean = 20.3 vs. WT FPI mean = 47.2, *p* = 0.004; day 4 IL-1α KO sham mean = 18.1 vs. IL-1α KO FPI mean = 40.5, *p* = 0.013). However, there was no difference between WT and IL-1α KO FPI groups on any of the days of training. In the training experiments involving IL-1β WT and KO littermates, the interaction of time × treatment was not significant (*F*_9,132_ = 1.095, *p* = 0.37), but the main effect of time was significant (*F*_3,132_ = 31.45, *p* < 0.0001). *Post hoc* testing reveled a difference in WT sham and FPI treatment groups on day 4 of training (latency to escape day 4 WT sham mean = 20.4 vs. WT FPI mean = 44.4, *p* = 0.03). Again, however, there was no difference between WT and IL-1β KO FPI groups on any of the days of training. In the Barnes maze training experiments involving IL-1RI WT and KO littermates, there was a significant interaction of time × treatment (*F*_9,165_ = 5.862, *p* < 0.0001). *Post hoc* testing showed differences in sham and FPI treatment groups (latency to escape day 4 WT sham mean = 24.5 vs. WT FPI mean = 58.2, *p* < 0.0001; day 4 IL-1RI KO sham mean = 25.7 vs. IL-1RI KO FPI mean = 49.5, *p* = 0.006), but no difference between WT and IL-1RI KO FPI groups on any of the days of training (Fig. 6).

While there was no effect of genotype on learning during the training phase of the Barnes maze, IL-1RI–deficient mice exposed to FPI performed significantly better than WT littermates in probe trial testing on day 5 (time in escape zone WT FPI mean = 1.95 s vs. IL-1RI–/– mean = 9.92 s, *p* = 0.01; Fig. 6). This is consistent with improvement in hippocampal-dependent spatial memory after TBI in the absence of IL-1RI signaling (Fig. 6*F*). This protective effect was not seen with specific IL-1α or IL-1β deficiency.

### Anakinra treatment after FPI decreases 24-h IL-1β expression in parietal cortex, but not in brainstem

**Figure 7.**
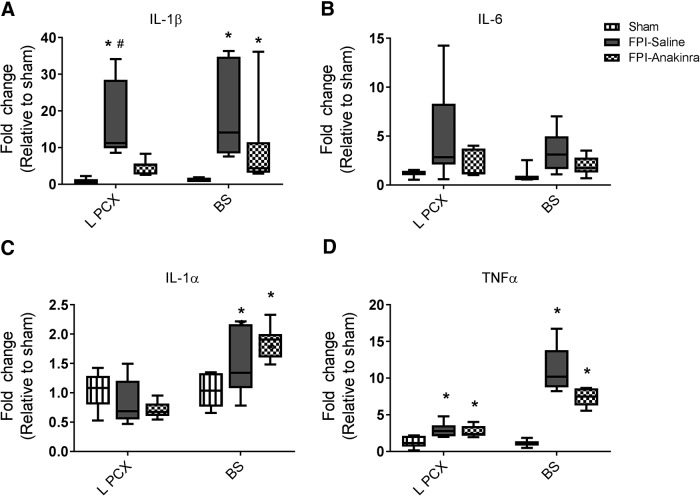
Anakinra treatment decreased regional IL-1β expression 24 h after FPI. Expression of IL-1β, IL-6, IL-1α, and TNF-α were evaluated by qPCR in ipsilateral parietal cortex (L PCX) and brainstem (BS) in FPI mice treated with anakinra (*n* = 7), 25 mg/kg IP twice daily for 1 d, or equal volume of normal saline (*n* = 8) and compared to untreated sham controls (*n* = 3). First dose of treatment (anakinra or saline) was initiated within 30 min of FPI, with second dose given ∼12 h later. Data are expressed as fold change in gene expression relative to sham and are presented as box-and-whisker plots; the box extends from 25th to 75th percentiles, the line represents the median, and the whiskers extend from smallest to largest value. One-way ANOVA with Dunn’s test for multiple comparisons. *, *p* < 0.05 compared with sham, #, *p* < 0.05 compared with FPI-anakinra. The data are pooled from two independent experiments.

Given the selective improvements in cytokine expression and cognitive functional outcome in IL-1RI–deficient mice that were not seen in either IL-1α– or IL-1β–deficient mice, we next sought to evaluate the effectiveness of selective pharmacologic inhibition of IL-1RI after FPI using anakinra, the FDA-approved recombinant form of a naturally occurring IL-1RI antagonist. By assessing the effects of pharmacologic blockade, we aimed to determine the therapeutic potential of acute IL-1RI blockade and address potential confounding effects of developmental perturbations that may occur in constitutive knockout models. Anakinra is a large, systemically administered molecule that is delivered at increased doses to improve CNS bioavailability. As such, we used a high dose of 25 mg/kg IP twice daily starting within 30 min after FPI and performed brain cytokine analysis 24 h post-FPI. Mice were randomly assigned to receive either anakinra or vehicle control. Quantitative PCR was used to assess cytokine expression 24 h after injury in the ipsilateral parietal cortex and in the brainstem to assess both directly contused tissue as well as tissue remote from impact. Twenty-four hours after injury, FPI resulted in a significant increase in IL-1β in the ipsilateral parietal cortex and brainstem, IL-1α in the brainstem, and TNFα in the ipsilateral parietal cortex and brainstem compared with sham mice. IL-6 was not significantly increased in either ipsilateral parietal cortex or brainstem and IL-1α was not significantly increased in the ipsilateral parietal cortex of FPI injured mice compared with sham mice, reflecting that expression of these cytokines resolves more quickly than the other cytokines evaluated. On assessing the impact of anakinra treatment on FPI-induced cytokine expression, we found that anakinra-treated mice exhibited decreased IL-1β expression in the ipsilateral parietal cortex compared with saline-treated FPI mice (Fig. 7*A*). In the brainstem, there was also a trend toward decreased IL-1β expression in anakinra-treated FPI mice (Fig. 7*A*). While IL-6 expression was not significantly increased by TBI in this experiment, a trend toward decreased expression in anakinra-treated versus saline-treated FPI subjects in both regions was noted (Fig. 7*B*). As expected, anakinra had no impact on IL-1α or TNFα expression in the ipsilateral parietal cortex or brainstem after FPI, consistent with our results demonstrating that IL-1RI deficiency also did not impact the expression of these cytokines after FPI (Fig. 7*C*, *D*).

### Anakinra treatment protects mice from FPI-induced cognitive dysfunction

**Figure 8.**
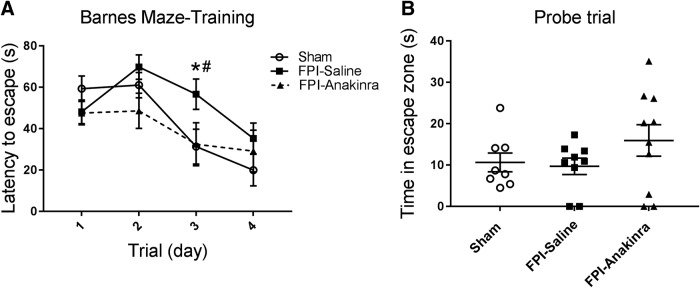
Effects of anakinra on cognitive function after FPI. Anakinra treatment resulted in improved learning on day 3 after FPI. After FPI, mice were treated with anakinra (*n* = 10; 25 mg/kg IP twice daily × 3 days) or normal saline (*n* = 9; equal volume twice daily × 3 days) and were compared to untreated shams (*n* = 8). On day of FPI, twice-daily treatment was initiated with first dose given within 30 min after FPI and second dose given 9 ± 1 h later. On days 1 and 2 post-injury, anakinra or saline was given every 12 h. Mice underwent Barnes maze testing beginning 2 wk post-injury. ***A***, ***B***, Spatial learning was assessed with 4 d of acquisition training (***A***), followed by a probe trial on day 5 (***B***) to assess short-term memory. Data are presented as mean ± SEM. Acquisition training two-way RM ANOVA with Fisher’s LSD for multiple comparisons. Probe trial one-way ANOVA with Fisher’s LSD for multiple comparisons. *, *p* < 0.05 FPI-saline compared with Sham, #, *p* < 0.05 FPI-saline compared with FPI-anakinra. The data are pooled from three independent experiments.

After observing that anakinra mitigated brain cytokine expression after FPI, we proceeded with functional evaluation as well. While peak IL-1β expression occurs in the first 24 h after TBI, expression continues at lower levels in the days after, leading us to extend anakinra treatment for 3 d in our behavioral studies. Cognitive function was assessed beginning 2 wk after FPI using the same paradigm of Barnes maze testing. Here, anakinra treatment resulted in more rapid learning in anakinra-treated FPI mice than in those receiving saline. During the training phase of Barnes maze testing, there was a significant interaction between time and treatment (*F*_6,72_ = 2.338, *p* = 0.04), and on *post hoc* testing, anakinra-treated FPI mice had significantly shorter latency to escape compared with saline-treated FPI mice on day 3 (latency to escape day 3 FPI-anakinra mean = 32.5 vs. FPI-saline mean = 56.7, *p* = 0.03). Additionally, while saline-treated FPI mice performed significantly worse than sham mice on day 3 of training, anakinra treated FPI mice showed no difference compared with sham mice (latency to escape day 3 sham mean = 31.3 vs. FPI-saline mean = 56.7, *p* = 0.03; latency to escape day 3 sham mean = 31.3 vs. FPI-anakinra mean = 32.5, *p* = 0.91; Fig. 8*A*). Anakinra-treated mice also showed a strong trend toward increased time in the escape zone on the day 5 probe test, relative to vehicle-treated animals that underwent FPI.

## Discussion

While it is well known that IL-1 signaling is a major mediator of secondary neurologic injury under many circumstances, the relative contributions of individual IL-1 pathway molecules to secondary injury after TBI have not been previously described. Given that FDA-approved IL-1 pathway antagonists already exist, however, dissecting the function of individual IL-1 pathway molecules on TBI outcome is essential for understanding the possible avenues of anti–IL-1 therapy for patients suffering from TBI. Here, we show for the first time that therapeutically targeting IL-1RI may be more likely to provide effective treatment for patients suffering cognitive deficits after TBI than drugs that specifically target individual IL-1α and IL-1β signaling. Specifically, we observed that genetic ablation of IL-1RI conferred a greater protective effect for cognitive function after TBI than did genetic ablation of either IL-1α or IL-1β. Moreover, this protective effect was recapitulated by treatment with the IL1-RI antagonist anakinra. Our data thus show that while IL-1α and IL-1β may have additive effects in TBI-induced injury, therapeutic blockade of both pathways through blocking their common receptor is more effective at preserving cognitive function after TBI. After TBI, cognitive dysfunction is common, with more than half of patients who sustain a moderate to severe TBI experiencing chronic cognitive impairment (Rabinowitz and Levin, 2014). Because the degree of cognitive dysfunction contributes significantly to lower quality of life, any treatment that improves cognition after TBI will have a great impact.

In this study, we have also shown that blocking the IL-1RI, but not individually blocking IL1-α or IL1-β signaling, protects mice from propagation of diffuse neuroinflammatory cytokine expression in the brain after TBI. This diffuse neuroinflammation may drive glial-mediated chronic axonal degeneration (Li et al., 1998; Kelley et al., 2006; Park et al., 2007; Sinha et al., 2017), and interrupting this process could thus help mitigate chronic cognitive deficits after TBI. In humans, focal TBI also results in diffuse brain inflammation and cognitive dysfunction. This suggests that the neuroinflammatory response in regions remote from the site of impact should be closely studied, and that our model system provides a relevant way to do this. Whether IL-1RI blockade might also provide benefit in chronic TBI injury will be the subject of future investigation in our lab, as there is a tremendous need for therapeutic support for patients who have suffered a TBI in the past and are no longer within the window for acute treatment.

At 6 h after injury, IL-1RI ablation did not prevent increased inflammatory cytokine expression at the injury epicenter, but it did prevent increased IL-1β and IL-6 in areas remotely located from the site of impact. This suggests that cytokine responses at the injury epicenter and diffuse regions are driven by different mechanisms. After moderate to severe TBI, necrotic cell death occurs rapidly at the site of focal injury, with resultant release of cellular contents that serve as endogenous danger signals activating local and invading immune cells (Dietrich et al., 1994). These signals include ATP, HMGB1, and S100B proteins, as well as cytokines IL-1α and IL-33 (Gadani et al., 2015; Bertheloot and Latz, 2017), which signal through a variety of receptors, including Toll-like receptors, Nod-like receptors, and the receptor for advanced glycation end products (RAGE). The net result is glial and invading immune cell activation and cytokine production (Gadani et al., 2015). It is thus not surprising that in areas of direct impact, IL-1RI deficiency alone was unable to prevent the early (6 h post-FPI) increase in inflammatory cytokine expression. In areas remote from impact, however, the primary mechanism of neuronal injury is diffuse axonal injury, whereby delayed axotomy is accompanied by limited, slowly progressive neuronal cell death (Singleton et al., 2002; Singleton and Povlishock, 2004). Here, IL-1RI ablation decreased the expression of two key inflammatory cytokines, IL-1β and IL-6. This highlights the need to consider different regional effects when assessing the impact of an intervention on the secondary inflammatory response, in both preclinical and clinical TBI studies.

Of note, while IL-1α deficiency did not impact cytokine expression 6 h after FPI in any of the regions examined, IL-1β deficiency did result in decreased IL-1α expression in the parietal cortex and brainstem, with a trend toward a decrease in the hippocampus. This was unexpected, as IL-1RI deficiency had no effect on IL-1α expression 6 h after FPI. Thus, this suggests that IL-1β can drive IL-1α expression in an IL-1RI–independent fashion. While IL-1RI is believed to be the primary receptor through which IL-1α and IL-1β signal, IL-1RI–independent effects in the CNS have been previously described (Qian et al., 2012). Like IL-1α deficiency, however, IL-1β deficiency had no impact on IL-6 expression, indicating that blockade of both cytokines is necessary to prevent the acute increase in IL-6, as was seen in IL-1RI–deficient mice. With respect to another inflammatory cytokine, TNFα, there was no impact on expression in the ipsilateral parietal cortex, ipsilateral hippocampus, or brainstem as a function of TBI in all 3 lines of knockout mice (IL-1α–, IL-1β–, and IL-1RI–deficient). However, IL-1RI–deficient mice did exhibit a significant decrease in TNFα expression in the cerebellum. The overall minimal impact on TNFα expression with interruption of IL-1 signaling suggests that pathways other than IL-1 play a greater role in controlling TNFα expression after TBI.

After TBI, the early development of inflammation occurs by mechanisms distinct from its maintenance, and cells initially activated by cell death and danger signals secrete inflammatory mediators, including cytokines that result in persistence of inflammation. Thus, in addition to evaluating the impact of IL-1RI deficiency on 6-h cytokine expression, we evaluated 24-h cytokine expression after TBI. IL-1 is one of the earliest cytokines released after immune cell activation, is known to trigger the expression of many other downstream inflammatory mediators, and may thus contribute significantly to ongoing immune cell activation after TBI. We report here for the first time that IL-1RI deficiency results in more rapid resolution of IL-1β and IL-6 levels at both the injury epicenter and remotely in the brainstem, confirming a critical role of IL-1 in perpetuating inflammation after TBI.

In addition to examining the impact of individual IL-1 pathway molecules on the inflammatory response to TBI, we were interested in determining the impact of these molecules on tissue loss after TBI. Despite the impact on cytokine expression, we found no evidence of cortical tissue protection with deficiency of IL-1RI or either IL-1 cytokine alone. In all three IL-1 pathway knockout lines, FPI-induced lesion size was similar to that seen in their WT littermates, suggesting that IL-1 signaling may not significantly influence cortical cell death at the site of impact. Rapid cell death occurs at the impact site, with a prior study demonstrating necrotic, irreversibly injured neurons as early as 1 h post-FPI (Hicks et al., 1996). Because peak IL-1 response may not occur for ≥2 h, IL-1 would be unlikely to play a significant role in early necrotic cell death that results in the cortical lesion after FPI (Kinoshita et al., 2002). A future direction will be to investigate delayed secondary cell death after diffuse axonal injury in our IL-1 pathway knockout lines of mice at chronic time points, to assess the impact of IL-1 on chronic neuronal death after TBI.

Given that IL-1RI deficiency had greater impact on inflammation and functional outcome than either IL-1α or IL-1β alone, we were interested in investigating whether this protective effect was replicated by treatment with anakinra, an FDA-approved IL-1RI antagonist. Treatment with anakinra after FPI revealed decreased IL-1β expression in the ipsilateral parietal cortex and a trend toward a decrease in the brainstem. This confirmed that pharmacologic IL-1RI blockade can at least partially achieve the attenuation of the cytokine response that was seen in genetic IL-1RI–deficient mice. The decreased impact of anakinra on brainstem cytokine expression, relative to genetic ablation of IL-1RI, may be due to limited CNS penetration. In unpublished work using IgG immunohistochemistry to evaluate blood brain barrier (BBB) leakage 24 h post-TBI, we noted that significant disruption of the BBB was limited to areas near the impact site, including the cortex and underlying hippocampus. Conversely, areas remote from impact, like the brainstem, did not show any evidence of BBB leakage by 24 h post-FPI. This is also consistent with previously published reports evaluating BBB integrity after FPI in rodent models (Hoshino et al., 1996). As anakinra is a large molecule, it has incomplete penetration into the CNS, with CNS levels characteristically only 2%–5% of serum levels (Gueorguieva et al., 2008; Fox et al., 2010; Greenhalgh et al., 2010; Galea et al., 2011). As such, the enhanced penetration that occurs in areas of significant BBB disruption may explain the more significant decrease in cytokine expression seen in the cortex near the site of injury, compared with the brainstem, after FPI. Overall, the decreased brain cytokine expression induced by anakinra treatment after FPI is encouraging, but the regional variation highlights the need for further studies to evaluate optimal dose and delivery methods.

Our functional outcome studies of mice treated with anakinra after FPI also revealed the potential for improving cognition after TBI. Anakinra-treated mice showed significant improvement in learning during the acquisition phase of the Barnes maze, compared with saline-treated FPI mice. While the limited CNS penetration of anakinra must be recognized, it is also known that systemic immune cells are activated and infiltrate the brain after TBI (Jin et al., 2012; Simon et al., 2017). Thus, systemically administered anakinra may exert some of its effects by modulating peripherally derived immune cells that infiltrate the brain after TBI. Our initial results showing brain cytokine modulation and improved cognitive function are encouraging and mandate further study to evaluate optimal dosing and delivery, as well as mechanistic studies examining the impact of IL-1 blockade on peripheral immune and CNS immune cells after TBI.

In conclusion, this study indicates the importance of blockade of both IL-1 cytokines to attain optimal protection after TBI. This can be achieved through blockade of IL-1RI, as shown by the use of IL-1RI–deficient mice as well as mice treated with the IL1-RI antagonist anakinra. Because the effect of anakinra only partially achieved the effects seen in IL-1RI–deficient mice, ongoing studies are needed to evaluate optimal dosing and delivery and determine the specific mechanism by which anakinra achieves its protective effects, including consideration of its impact on both peripheral and central immune responses.

## Synthesis

Reviewing Editor: Jun Chen, University of Pittsburgh Decisions are customarily a result of the Reviewing Editor and the peer reviewers coming together and discussing their recommendations until a consensus is reached. When revisions are invited, a fact-based synthesis statement explaining their decision and outlining what is needed to prepare a revision will be listed below. The following reviewer(s) agreed to reveal their identity: Jonathan Godbout, Dong Sun

Both reviewers believe that their previous concerns have been addressed and the manuscript is improved. However, Reviewer #2 suggested that “the new fig. 4 about the righting time is unnecessary to be included”.

## Author Response

As noted below, we have made the requested edits to our manuscript in response to the reviewers' comments.

Synthesis Statement for Author (Required):

Both reviewers believe that their previous concerns have been addressed and the manuscript is

improved. However, Reviewer #2 suggested that “the new fig. 4 about the righting time is unnecessary to be included”.

We have removed figure 4 and the corresponding text and renumbered the other figures accordingly.
